# AcT-2: A Novel Myotropic and Antimicrobial Type 2 Tryptophyllin from the Skin Secretion of the Central American Red-Eyed Leaf Frog, *Agalychnis callidryas*

**DOI:** 10.1155/2014/158546

**Published:** 2014-02-13

**Authors:** Lilin Ge, Peng Lyu, Mei Zhou, Huiling Zhang, Yuantai Wan, Bin Li, Renjie Li, Lei Wang, Tianbao Chen, Chris Shaw

**Affiliations:** Natural Drug Discovery Group, School of Pharmacy, Queen's University, Belfast BT9 7BL, UK

## Abstract

Tryptophyllins are a diverse family of amphibian peptides originally found in extracts of phyllomedusine frog skin by chemical means. Their biological activities remain obscure. Here we describe the isolation and preliminary pharmacological characterization of a novel type 2 tryptophyllin, named AcT-2, from the skin secretion of the red-eyed leaf frog, *Agalychnis callidryas*. The peptide was initially identified during smooth muscle pharmacological screening of skin secretion HPLC fractions and the unique primary structure—GMRPPWF-NH_2_—was established by both Edman degradation and electrospray MS/MS fragmentation sequencing. A. cDNA encoding the biosynthetic precursor of AcT-2 was successfully cloned from a skin secretion-derived cDNA library by means of RACE PCR and this contained an open-reading frame consisting of 62 amino acid residues with a single AcT-2 encoding sequence located towards the C-terminus. A synthetic replicate of AcT-2 was found to relax arterial smooth muscle (EC_50_ = 5.1 nM) and to contract rat urinary bladder smooth muscle (EC_50_ = 9.3 **μ**M). The peptide could also inhibit the growth of the microorganisms, *Staphylococcus aureus*, (MIC = 256 mg/L) *Escherichia coli* (MIC = 512 mg/L), and *Candida albicans* (128 mg/L). AcT-2 is thus the first amphibian skin tryptophyllin found to possess both myotropic and antimicrobial activities.

## 1. Introduction

Amphibians represent the most ancient group of terrestrial vertebrates and are widely distributed globally, occurring on all continents with the exception of Antarctica [1, 2]. Their continuing persistence in the biosphere can in part be attributed to their highly developed and potent skin secretions that act as a front-line defense against potential predators and microbes [1, 2]. The biological potency of their skins and associated secretions were recognized by many ancient peoples who used such in shamanic rituals and as medicines for diverse ailments [1, 2]. Some applications of these secretions include those of bufonid toads as stimulants in traditional Chinese medicine, those of tropical poison frogs (dendrobatids) as arrow-tip poisons, and those of giant monkey frogs (*Phyllomedusa bicolor*) in “hunting magic”—a purging and sensory-enhancing ritual performed by some South-American natives prior to hunting [3].

The unlocking of the molecular secrets of amphibian defensive skin secretions was predominantly initiated by two, twentieth-century pioneer pharmacological chemists—Vittorio Erspamer (1909–1999) (endogenous peptides and biogenic amines) and John Daly (1933–2008) (exogenous alkaloids). Their combined efforts resulted in the unravelling of the molecular structures and biological actions of several thousands of such molecules representing many classes of biochemical, studies on which have served to reveal many novel regulatory systems and potential drug targets in mammalian systems [1–4].

The application of modern analytical technologies such as molecular cloning, proteomics, and mass spectrometry to the study of the molecular complexity of amphibian skin secretions continues on where the pioneers left off with ever-increasing numbers of unique molecules of diverse functions being revealed [5]. One of the major goals of such contemporary research is directed towards the identification of natural leads for therapeutic development or to identify novel disease-related drug targets [6, 7].

The bioactive peptides represent one of the largest and most functionally diverse groups of biochemicals identified thus far from amphibian skin, and unlike the diet-acquired alkaloids, these are of endogenous origin and are genetically encoded [1–4]. While many individual peptides have been isolated, they fall into two broad functional groupings—those with pharmacological activity and those with antimicrobial activity [1–4].

Tryptophyllins (TPHs) are a large and heterogenous group of peptides that did not fall into either category when first identified, as they were originally isolated from the skin of the South-American leaf frog, *Phyllomedusa rohdei*, through chemical means by nature of their positive Ehrlich's reaction—a color-producing chemical reaction for indoles (tryptophan residues) [2]. All original TPHs possessed a tryptophanyl residue at position 2 from the C-terminus and prolyl residues at position 2 and/or 3 from the N-terminus [2], although some TPHs more recently identified are devoid of tryptophanyl residues [2]. Due to the increasing numbers of TPHs being identified and their apparent structural heterogeneity, this peptide family has been divided into three types—(1) tryptophyllin-1 (T-1) peptides: the N-terminal doublet KP-, a tryptophanyl residue at position 5 and a prolyl residue at position 7 from the N-terminus; are all highly conserved in this group of peptides that typically possess 7-8 residues; (2) tryptophyllin-2 (T-2) peptides: these are variable in length, contain 4 to 7 residues, and all contain an internal-PW doublet; (3) tryptophyllin-3 (T-3) peptides: the most conserved TPHs containing 13 residues with conservative substitutions at positions 2, 5, 6, and 13 from the N-terminus. The N-terminal pGlu-, -KP-, at positions 3 and 4 and the 7–12 sequence, -PPPIYP-, are all completely conserved [2, 8–10].

Although the structures of many TPHs have been known for more than twenty-five years, their biological actions have remained somewhat obscure until recently. In early reports, TPHs were described as having some general metabolic effects, such as stimulating liver protein synthesis and antibodies raised to several of these peptides, produced positive immunostaining in pituitary gonadotrophs [2]. In more recent times, two tryptophyllins (PdT-1 and PdT-2) from the skin secretion of the Mexican leaf frog, *Pachymedusa dacnicolor*, were found to have potent pharmacological effects on different rat smooth muscles [8, 9].

Here, we describe the identification, structural, and functional characterization of a novel Type-2 tryptophyllin, named AcT-2, from the skin secretion of the Central American red-eyed leaf frog, *Agalychnis callidryas*. This peptide was first identified through pharmacological screening for myotropic activity and was subsequently, in addition, found to have a broad spectrum of antimicrobial activity, albeit relatively low potency. Unusually, the peptide was found to possess a higher potency against the yeast, *Candida albicans*, than against the model Gram-positive and Gram-negative bacteria employed.

## 2. Materials and Methods

### 2.1. Acquisition of Skin Secretions

Adult red-eyed tree frogs, *Agalychnis callidryas* (*n* = 6), of the Costa Rican type, were housed in a purpose-designed terrarium under a 12 h/12 h light/dark cycle and were fed multivitamin-loaded crickets three times per week. Skin secretions were obtained by transdermal electrical stimulation (4 ms pulse width, 50 Hz, 6 V) in accordance with the method of Tyler et al. [11] following a three-month settling-in period. The skin secretions were washed from the skin using deionized water, snap frozen in liquid nitrogen, lyophilized, and stored at −20°C prior to analyses.

### 2.2. Reverse Phase HPLC Fractionation of Lyophilized Skin Secretion

Five milligrams of lyophilized skin secretion were dissolved in 1 mL of 0.05/99.95 (v/v) trifluoroacetic acid (TFA)/water and clarified by centrifugation. The supernatant was directly injected onto a CECIL reverse phase HPLC system (Milton Technical Centre, Cambridge, UK) fitted with an analytical column (Phenomenex Jupiter C5; 300 Å pore size; 250 × 4 mm). The linear elution gradient employed was formed from 0.05/99.95 (v/v) TFA/water to 0.05/19.95/80.0 (v/v/v) TFA/water/acetonitrile in 80 min at a flow rate of 1 mL/min. The effluent absorbance was monitored at *λ* = 214 nm and fractions of approximately 1 mL were collected automatically at minute intervals. Samples (100 *μ*L) from each chromatographic fraction were removed, lyophilized, and stored at −20°C prior to analysis for myoactivity using rat smooth muscle bioassays.

### 2.3. Rat Smooth Muscle Bioassays

Male Wistar rats (250–300 g) were euthanized by carbon dioxide asphyxiation followed by cervical dislocation under appropriate UK animal research lisences and following local institutional guidelines. After removing the fur on the abdomen of rats, the body cavity was opened and any visible fat was removed. The exposed urinary bladder and the tail were carefully removed. All dissected tissues were placed in ice-cold Kreb's solution (118 mM NaCl, 4.7 mM KCl, 25 mM NaHCO_3_, 1.15 mM NaH_2_PO_4_, 2.5 mM CaCl_2_, 1.1 mM MgCl_2_, and 5.6 mM glucose) which was aerated with 95% O_2_/5% CO_2_ gas mixture. Strips of urinary bladder and rings of dissected tail artery were prepared and tied with fine silk ligatures (0.2 mm) at each end. These preparations were attached to a fixed pin at one end and to a transducer at the other end. Kreb's solution (at 37°C) flowing through the organ baths at 2 mL/min with a constant bubbling of 95% O_2_/5% CO_2_ maintained the tissues in a viable state for >2 h. An equilibration period of 20 min was used for each preparation after which 60 mM KCl was added to test the responsiveness of urinary bladder muscle strips and 10^−3^ M phenylephrine was added to test the responsiveness of the tail artery rings and to cause preconstriction. Following these tests, viable preparations were used to screen prepared samples of reverse phase HPLC fractions of *Agalychnis callidryas* skin secretion. Changes in tension of smooth muscle preparations were detected by pressure transducers connected to a PowerLab System (AD Instruments Pty Ltd.). Data were analyzed using GraphPad Prism software and data points were expressed as mean values ± standard errors, *n* = 6 in each case.

### 2.4. Structural Characterization of the Novel Peptide

A single reverse phase HPLC fraction was found to have a potent relaxation effect on tail artery smooth muscle, a less potent contractile action on urinary bladder smooth muscle strips and a moderately potent antimicrobial effect. A sample of this fraction was subjected to MALDI-TOF mass spectrometry using a Perseptive Biosystems Voyager DE mass spectrometer in positive detection mode with *α*-cyano-4-hydroxycinnamic acid as matrix. Subsequent to this analysis, the single major peptide resolved was subjected to MS/MS fragmentation sequencing using an LCQ Fleet electrospray ion-trap mass spectrometer (Thermo-Fisher, San Jose, CA, USA).

### 2.5. Molecular Cloning of the Novel Peptide Biosynthetic Precursor-Encoding cDNA

Five milligrams of lyophilized skin secretion were dissolved in 1 mL of cell lysis/mRNA protection buffer that was supplied by Dynal Biotech, UK. Polyadenylated mRNA was then isolated from this using magnetic oligo-dT Dynabeads (Dynal Biotech, UK), as per manufacturer's instructions. The isolated polyadenylated mRNA was then subjected to 5′- and 3′-rapid amplification of cDNA ends (RACE) procedures to obtain full-length novel peptide precursor nucleic acid sequence data using a SMART-RACE kit (Clontech UK) likewise as per manufacturer's instructions. Briefly, the 3′-RACE reactions employed a nested universal (NUP) primer (supplied with the kit) and a degenerate sense primer (SP: 5′-GGIATGMGICCICCITGG-3′) (I = deoxyinosine, M = A/C) that was complementary to the putative amino acid sequence, GMRPPW-, of the novel peptide. The 3′-RACE reactions were purified and cloned using a pGEM-T vector system (Promega Corporation) and sequenced using an ABI 3100 automated sequencer. The sequence data obtained from the 3′-RACE product were used to design a specific antisense primer (ASP: 5′-CGGCACTATTACTGATAATTGTGCT-3′) to a defined site within the 3′ nontranslated region of the novel peptide precursor-encoding transcripts. 5′-RACE was carried out using these primers in conjunction with the NUP primer and resultant products were purified, cloned, and sequenced.

### 2.6. Solid-Phase Peptide Synthesis

Once the unequivocal primary structure of the novel peptide had been established, it was chemically synthesized by solid-phase Fmoc chemistry using a PS3 automated solid-phase peptide synthesizer (Protein Technologies, Inc., AZ, USA). Following cleavage of the synthetic peptide from the resin and deprotection of the side-chain protecting groups, the resultant material was lyophilized and then purified by HPLC. The major product was subjected to MALDI-TOF mass spectrometry to establish both degree of purity and authenticity of structure.

### 2.7. Preliminary Pharmacological Characterization of the Synthetic Novel Peptide on Rat Tail Artery and Urinary Bladder Smooth Muscle

The synthetic peptide was initially prepared as a stock in Kreb's solution (118 mM NaCl, 4.7 mM KCl, 25 mM NaHCO_3_, 1.15 mM NaH_2_PO_4_, 2.5 mM CaCl_2_, 1.1 mM MgCl_2_, and 5.6 mM glucose) at a concentration of 10^−3 ^M. Working concentrations of peptide, ranging from 10^−3^ to 10^11^ M, were prepared prior to each experiment and were applied to tissues (*n* = 6) progressively from low to high concentrations. Data obtained from the PowerLab System (AD Instruments Pty, Ltd.) were analyzed by Student's *t*-test through GraphPad Prism software to obtain the mean and standard error of responses and using these datasets, dose-response curves were constructed using a best-fit algorithm.

### 2.8. Determination of Minimal Inhibitory Concentrations (MICs) of the Synthetic Novel Peptide for Model Microorganisms

Minimal inhibitory concentrations (MICs) of the synthetic peptide were assessed against three standard model microorganisms: the Gram-positive bacterium *Staphylococcus aureus *(*S. aureus*, NCTC 10788), the Gram-negative bacterium *Escherichia coli *(*E. coli*, NCTC 10418) and the pathogenic yeast *Candida albicans *(*C. albicans,* NCPF 1467). The synthetic peptide was initially dissolved in a small volume of dimethylsulfoxide (DMSO) and made up to 1 mL with sterile Muellar-Hinton broth (MHB). Doubling dilutions of this peptide stock solution peptide were made from 512–1 mg/L in sterile MHB and were incubated with microorganism cultures (10^6^ colony forming units (CFU)/mL) in 96-well microtiter cell culture plates for 18 h at 37°C, in a humidified atmosphere. MHB alone was used as a negative control and each microorganism in MHB with no peptide added was used as positive controls. After incubation, the growth of microorganisms was determined by means of measuring optical density (OD) at *λ* = 550 nm using an ELISA plate reader (Biolise BioTek EL808). Minimal inhibitory concentrations (MICs) were defined as the lowest concentration at which no growth was detectable.

### 2.9. Hemolysis Assay

Peptide solutions in a range of concentrations were prepared as described in Section 2.8 but in 0.9% (w/v) aqueous NaCl solution, prior to performing the hemolysis assay. A 2% suspension of washed horse red blood cells in this solution was prepared from defibrinated horse blood (TCS Biosciences Ltd., UK) and samples of this were incubated at 37°C for 120 min, with a range of peptide concentrations similar to those employed for the antimicrobial assays. Lysis of red cells was assessed by measurement of the optical density of supernatants following centrifugation at *λ* = 550 nm using an ELISA plate reader (Biolise BioTek EL808). Negative controls employed consisted of a 2% (v/v) red cell suspension alone and positive controls consisted of a 2% (v/v) red cell suspension and an equal volume of saline containing 2% (v/v) of the nonionic detergent, Triton X-100 (Sigma-Aldrich).

## 3. Results

### 3.1. Identification and Structural Characterization of the Novel Peptide

**Figure 1:**
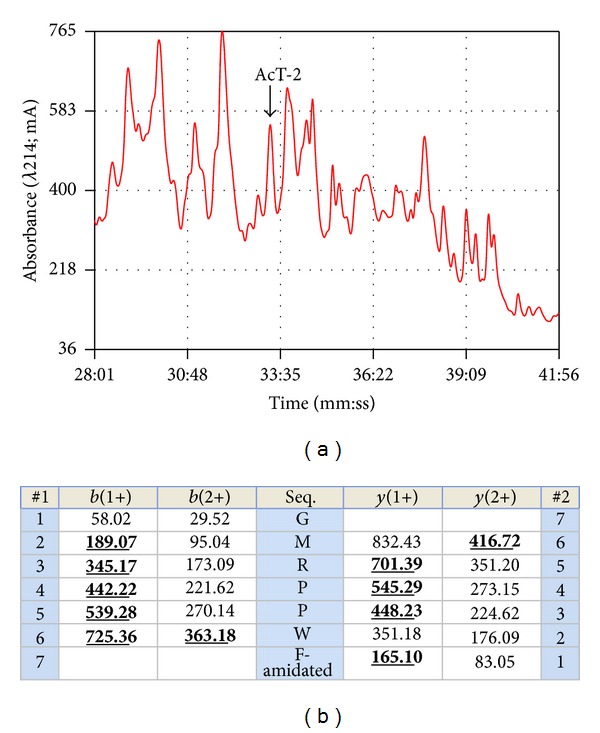
Region of reverse phase HPLC chromatogram of *Agalychnis callidryas* skin secretion indicating the elution position/retention time (arrow) of absorbance peak containing the peptide, AcT-2 (a). Expected singly and doubly charged *b-*ion and *y-*ions arising from fragmentation of AcT-2 as predicted using the MS-Product program available through Protein Prospector online. Observed fragment ions are indicated in bold type face and are underlined (b).

**Figure 2:**
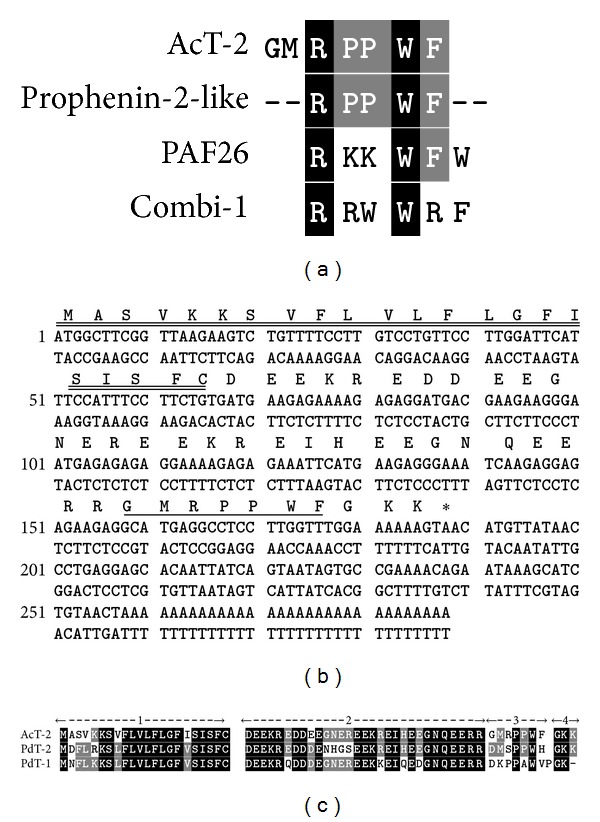
Comparison of the primary structure of AcT-2 with those of similar peptides. PAF26 and combi-1 are synthetic antifungal peptides isolated from combinatorial peptide libraries [12, 13]. Prophenin-2-like is a region of an antimicrobial polypeptide, cathelicidin, sequenced from a genomic DNA template of the killer whale (*Orcinus orca*) (accession no. XP004284013). Conserved amino acid residues are shaded black and consensus residues are shaded grey (a). Nucleotide and translated open-reading frame amino acid sequence of a cloned cDNA encoding the biosynthetic precursor of AcT-2. The putative signal peptide is double-underlined, the mature peptide is single-underlined and the stop codon is indicated by an asterisk (b). Alignments of complete open-reading frame amino acid sequences of AcT-2 precursor-encoding cDNA with the two top hits obtained following BLAST analysis using the NCBI portal. PdT-1 and PdT-2 represent *Pachymedusa dacnicolor* tryptophyllin-1 and -2, respectively. Conserved amino acids are shaded black and consensus amino acids are shaded grey. Gaps have been inserted to maximize alignments. (1) Putative signal peptide domain. (2) Acidic spacer peptide domain. (3) Mature peptide domain. (4) C-terminal processing site (c).

Reverse phase HPLC fraction #33 of *Agalychnis callidryas* skin secretion (Figure 1(a)) was found to contain a myoactive peptide following preliminary smooth muscle pharmacological screening and a sample of this fraction was subjected to MALDI-TOF mass spectrometry which indicated a relatively high degree of purity of a peptide with an *m/z* (M+H)^+^ of 889.25. The doubly charged ion of this peptide was subsequently identified following electrospray MS analysis and subjected to MS/MS fragmentation sequencing (Figure 1(b)). This produced the tentative amino acid sequence, GMRPPWF, based upon identification of *b-* and *y-*ion series. The peptide was also deemed to be C-terminally amidated. Bioinformatic analysis produced no hits with any archived amphibian skin peptide but with two synthetic antifungal peptides, named PAF-26 and combi-1 [12, 13] (Figure 2(a)). Of note was the presence of residues 3–8 of the novel myotropic peptide in a C-terminally-located domain of a prophenin-2-like protein from the killer whale (*Orcinus orca*) that contains a cathelicidin sequence (accession no. XP004284013) (Figure 2(a)). However, the origin and structural characteristics of the novel myotropic peptide, an internal -PPW- sequence and C-terminal amidation, indicated that it was a member of the amphibian skin tryptophyllin family, subtype T-2, and thus it was named *Agalychnis callidryas* tryptophyllin-2 (AcT-2) in accordance.

### 3.2. Molecular Cloning of AcT-2 Biosynthetic Precursor-Encoding cDNA

A cDNA encoding the AcT-2 precursor protein was successfully and repeatedly cloned using the RACE PCR strategy employed. The sequence was represented in at least 30 clones after employing repetitive PCR and cloning procedures. The complete nucleotide and translated open-reading frame amino acid sequences of the cloned AcT-2 biosynthetic precursor-encoding cDNA are shown in Figure 2(b). The deduced open-reading frame consisted of 62 amino acid residues and shared a similar architecture with the previously reported precursors of the tryptophyllins, PdT-1 and PdT-2 from *Pachymedusa dacnicolor* skin secretion (Chen et al. 2004, Wang et al. 2009) (Figure 2(c)). This consisted of an N-terminal putative signal peptide, an acidic amino acid residue-rich spacer peptide domain, a single copy of a mature AcT-2 sequence, and a C-terminal processing and amidation site (Figure 2(c)). As in the *Pachymedusa dacnicolor* tryptophyllin precursors, the mature peptide was flanked N-terminally by a double basic amino acid residue propeptide convertase processing site - -RR-, cleavage of which generated the N-terminus of mature AcT-2. The C-terminal region of the mature AcT-2 peptide was flanked by a tripeptide sequence, -GKK, in common with the precursor of PdT-2. The double basic amino acid motif, -KK, is removed by propeptide convertase and the resulting C-terminal glycyl (G) residue serves as an amide donor through the action of amidating enzyme complex.

### 3.3. Smooth Muscle Activity of Synthetic AcT-2

**Figure 3:**
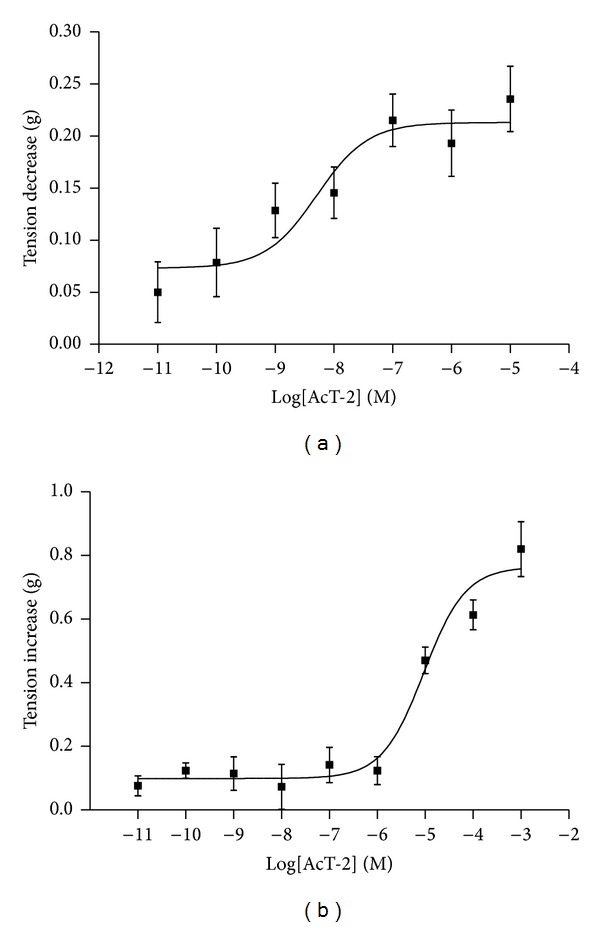
Dose-response curves obtained using synthetic AcT-2 on rat smooth muscle preparations. (a) Effect of the peptide on tail artery smooth muscle preparations expressed as means and standard errors (*n* = 6). EC_50_ was found to be 5.1 nM. (b) Effect of the peptide on urinary bladder smooth muscle preparations expressed as means and standard errors (*n* = 6). EC_50_ was found to be 9.3 *μ*M.

Synthetic AcT-2 was found to be active in smooth muscle preparations from both rat tail artery and urinary bladder but in different ways and at different potencies. In rat tail artery smooth muscle preparations, AcT-2 caused a dose-dependent relaxation with an EC_50_ of 5.1 nM (Figure 3(a)). In contrast, the peptide induced a dose-dependent contraction of urinary bladder smooth muscle with an EC_50_ of 9.3 *μ*M (Figure 3(b)).

### 3.4. Antimicrobial and Hemolytic Activity of AcT-2

**Figure 4:**
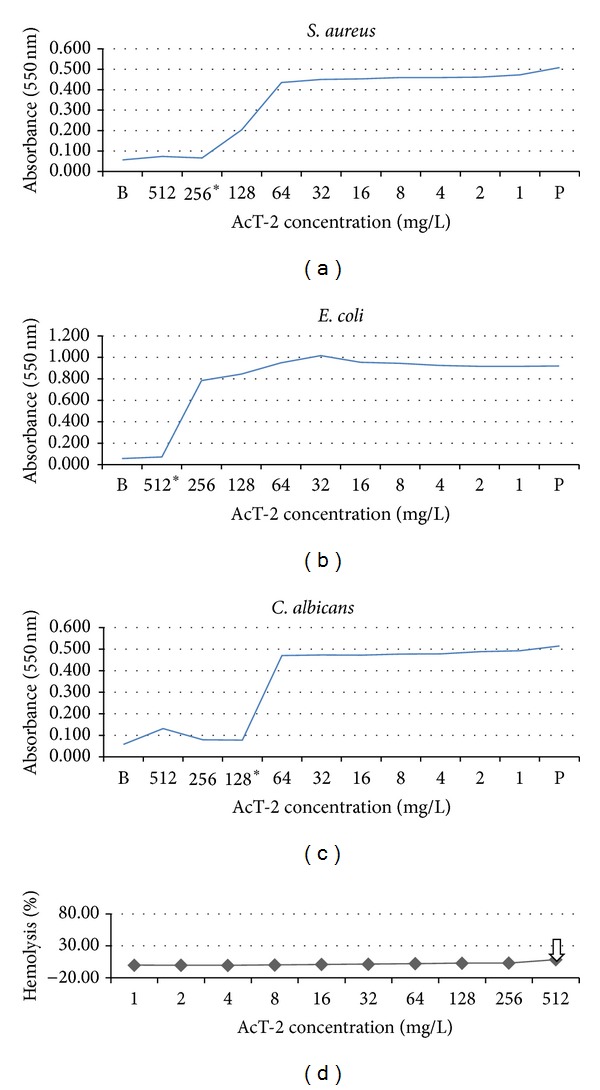
Minimal inhibitory concentration (MIC) curves obtained following incubation of synthetic AcT-2 with *S. aureus* (a), *E. coli* (b), and *C. albicans* (c). The MIC value for each microorganism is indicated in respective panels by asterisks. (d) The hemolytic activity of synthetic AcT-2. The arrow shows the highest concentration of peptide (512 mg/L) which was the only concentration at which hemolysis was observed (8.5%).

AcT-2 was somewhat unexpectedly found to possess a broad spectrum of antimicrobial activity. MICs obtained with the three model test organisms were as follows: *S. aureus* (256 mg/L), *E. coli* (512 mg/L), and *C. albicans* (128 mg/L) (Figures 4(a)–4(c)). The order of sensitivity of the test organisms employed was thus *C. albicans > S. aureus > E. coli, *which is a most unusual profile for previously reported typical amphibian skin cationic, amphipathic, *α*-helical antimicrobial peptides. Also worthy of note was the virtual lack of hemolytic activity of AcT-2, even up to the highest concentration (512 mg/L) employed (Figure 4(d)).

## 4. Discussion 

Central-American red-eyed leaf frogs* (Agalychnis callidryas) *are probably the most universally recognized frogs in the world having been used extensively by advertising agencies due to their strikingly beautiful colors. Although they are one of the most widely available of the phyllomedusine leaf frogs, studies on their defensive skin secretion peptides have not been carried out with the same degree of focus that has been given to many of their more obscure relatives [2, 14]. Red-eyed leaf frogs are most vividly colored with vibrant lime-green dorsal surfaces, white ventral surfaces, cream and cobalt-blue striped sides, and bright orange feet. Despite this, frogs are difficult to see as they press themselves against leaves and pull their limbs tightly towards their bodies, thus becoming a green blob on the leaf surface. Their bright coloration, however, is only present during the day when the frogs are largely inactive, while, during the hours of darkness, whenever the frogs are most active, their colors are much drabber favouring shades of grey and brown [15].

The potent cocktails of defensive molecules present in skin secretions effectively constitutes their front-line of defense against predators [1, 2]. To date, several peptides with a range of biological effects have been reported from these skin secretions and these include both antimicrobial and pharmacologically active peptides [1, 2, 16]. However, AcT-2 described here represents the first skin secretion tryptophyllin described from this species despite several having been reported from *Phyllomedusa* species over the past decades [2]. A recent report by Wang et al. [9] described a simple rational scheme to classify the rather structurally-heterogenous group of amphibian skin peptides collectively named tryptophyllins [9]. In this, peptides were classified into one of three groups, named T-1, T-2 and T-3 tryptophyllins, based on some common structural features. On this basis, AcT-2 shows the features of a type-2 (T-2) tryptophyllin, in common with PdT-2 [9], as both possess an internal -PW- sequence motif (Figure 2(c)). However, despite the presence of this common internal structural feature, AcT-2 and PdT-2 display radically different potencies in stimulating the contraction of rat uterus smooth muscle (EC_50_ values: AcT-2 = 9.3 *μ*M; PdT-2 = 4 nM), indicating that other features of the primary structure influence their receptor interactions [9]. However, AcT-2 was found to possess a most potent arterial smooth muscle relaxant effect (EC_50_ = 5.1 nM)—an effect not observed with PdT-2 [9]. The smooth muscle pharmacology and molecular targets for these Type-2 tryptophyllins in mammalian tissues obviously warrant further in-depth investigation.

As demonstrated in this study, AcT-2 was unexpectedly found to possess antimicrobial activity, albeit relatively low potency, using three model test microorganisms with the order of sensitivity being *C. albicans > S. aureus > E. coli* or in other words, yeast > Gram-positive bacterium > Gram-negative bacterium. Related to this observation was that following database interrogation with the primary structure of AcT-2, three peptides were found which displayed some degree of structural identity (Figure 2(a)). Two of these, named PAF26 and combi-1, were synthetic in nature and were found through the screening of combinatorial peptide libraries for antifungal activity [12, 13]. The third represented a cathelicidin domain of a prophenin-2-like protein deduced from a cloned cDNA of the killer whale (*Orcinus orca*) (accession no. XP004284013). Interestingly, all three AcT-2-like peptides were associated with antimicrobial functions, with the former two being specifically against fungi.

While AcT-2 is of relatively low potency when compared with many other amphibian skin antimicrobial peptides, most if not all of the latter act through a mechanism of microbial target cell membrane lysis and some are strongly hemolytic [17–19]. To achieve this form of nonspecific antimicrobial action, such peptides are generally much longer in amino acid chain length than AcT-2 [17, 19]. Thus, AcT-2 may act upon a different and possibly intracellular target rather than by direct lytic action on the cytoplasmic membrane—a mode of action that has actually been proposed for some “classical” antimicrobial peptides as well [17, 19]. In support of this proposal to some degree is the lack of hemolytic activity of AcT-2 observed following its incubation with horse erythrocytes (Figure 4(d)).

In conclusion, the novel amphibian skin tryptophyllin, named AcT-2, described here, has been demonstrated to have both selective mammalian smooth muscle myotropic effects and antimicrobial activity—findings which add to our increasing knowledge of the biological effects of this enigmatic family of amphibian skin peptides.

The nucleotide sequence of AcT-2, from the skin secretion of *Agalychnis callidryas*, has been deposited in the EMBL Nucleotide Sequence Database under the accession code HG710094.
